# Robot-Assisted Hysterectomy for Endometrial Cancer—Own Observations

**DOI:** 10.3390/jcm15083008

**Published:** 2026-04-15

**Authors:** Anna Bogaczyk, Tomasz Zuzak, Patryk Jasielski, Michał Maźniak, Andrzej Wróbel, Jan Wróbel, Marcin Misiek, Krzysztof Przyśliwski, Aleksander Rycerz, Tomasz Kluz

**Affiliations:** 1 Department of Gynecology, Gynecological Oncology and Obstetrics, Fryderyk Chopin University Hospital, 35-055 Rzeszow, Poland; annabogaczyk@interia.pl (A.B.); patryk.jasielski111@gmail.com (P.J.); m.mazniak@gmail.com (M.M.); jtkluz@interia.pl (T.K.); 2 Faculty of Medicine, University of Rzeszow, 35-959 Rzeszow, Poland; 3 Second Department of Gynecology, Medical University of Lublin, 20-090 Lublin, Poland; wrobelandrzej@yahoo.com; 4 Medical Faculty, Medical University of Lublin, 20-090 Lublin, Poland; wrobeljan@onet.eu; 5 Department of Gynecologic Oncology, Holy Cross Cancer Centre, 25-377 Kielce, Poland; mmisiek@me.com; 6 Faculty of Health Sciences and Psychology, University Center for Research and Development in Health Sciences, Collegium Medicum, University of Rzeszow, 35-959 Rzeszow, Poland; kprzysliwski@ur.edu.pl; 7 Medicover Wilanow Hospital, Medicover Healthcare Services Warsaw, 02-972 Warszawa, Poland; 8 Department of Surgical and Oncologic Gynecology, 1st Department of Gynecology and Obstetrics, Medical University of Lodz, Poland, 92-213 Lodz, Poland; aleksander.rycerz@umed.lodz.pl; 9 Department of Biostatistics and Translational Medicine, Medical University of Lodz, 92-215 Lodz, Poland

**Keywords:** endometrial cancer, robotic surgery, minimally invasive surgery

## Abstract

**Background:** Endometrial cancer is one of the most common cancers in women. In recent years, minimally invasive methods such as laparoscopy and robotic surgery have become very popular. Robotic surgery is a rapidly evolving and continuously improving modality. **Methods:** The main goal of our study was to compare patients operated on with the da Vinci robot with laparoscopy. The study included 300 patients with endometrial cancer who underwent surgery using the da Vinci robotic system and 80 patients with endometrial cancer who underwent laparoscopic surgery. **Results:** We have demonstrated that robot-assisted surgery is associated with significantly lower blood loss and a reduced risk of complications, whereas operative time remains shorter with laparoscopy. At the same time, we have observed that prolonged robotic operative time occurs particularly in older patients and those with a higher BMI, which should be taken into account when planning surgical procedures. **Conclusions:** Further research is needed to better define the groups of patients who benefit most and to optimize surgical strategies.

## 1. Introduction

Endometrial cancer (EC) is the sixth most frequently diagnosed cancer in women worldwide, and the second most common cancer of the female reproductive organs. Its incidence is increasing [1]. The highest incidence of EC is reported in North American and European countries, where it is the most common female genital cancer and the fifth most common cancer site in women after breast, lung, colon, and non-basal skin cancers [2]. High-income countries have a higher incidence of endometrial cancer (11.1/100,000 women) than low-income countries (3.3/100,000 women). This results from high rates of obesity, physical inactivity, an aging population, and increased life expectancy. High estrogen levels are considered the most likely cause of the increased risk of endometrial cancer in obese postmenopausal women [3]. Obesity is also associated with an earlier age at diagnosis of EC and endometrioid type, which is interesting since similar associations have not been observed in non-endometrioid cancers (different carcinogenesis pathways) [4]. The preventive factors of EC include physical activity and long-term use of continuous combined estrogen–progestogen therapy [5].

The diagnosis of EC is made based on histopathological findings obtained by endometrial aspiration biopsy or curettage, with or without hysteroscopy. The combination of transvaginal ultrasound and endometrial biopsy obtained by curettage has been shown to have a negative predictive value (NPV) of 96% [6].

The stage assessment is particularly useful in patients with suspected advanced disease and non-endometrioid histology. In patients with EC, imaging of the chest, abdomen, and pelvis based on computed tomography or PET-CT can help determine the surgical approach. MRI, on the other hand, allows an assessment of the depth of tumor invasion [7,8].

The standard surgical treatment is a complete simple hysterectomy, including oophorectomy and salpingectomy. Patients with FIGO stage I and II disease, as assessed clinically and radiologically, undergo surgery. Peritoneal fluid is not collected for cytological examination [9,10]. Since minimally invasive surgery (MIS), i.e., laparoscopic treatment and the treatment assisted by the robot, has a definite advantage over the classic laparotomy approach, it is recommended to perform surgery using MIS whenever possible [11,12]. MIS is the standard of care for endometrial cancer and is currently widely used in the treatment of endometrial cancer. It offers many advantages, including faster recovery and a shorter hospital stay. However, when introducing new surgical techniques in the context of treating cancer patients, it is essential to prioritize oncological efficacy and safety.

Robotic-assisted surgery in the treatment of patients with early-stage endometrial cancer is associated with favorable surgical and oncological outcomes. This is particularly true for patients from groups at higher surgical risk, such as older and obese women [13,14].

In this article, we have presented an evaluation of 300 patients undergoing surgery with the da Vinci robot for endometrial cancer. We have compared the robotic procedure with the laparoscopic approach. All patients underwent surgery at the same center but at different times. The vast majority of laparoscopic procedures were performed before the introduction of robotic surgery at our center.

We have highlighted the role and significant benefits for patients undergoing robotic surgery, as well as discussed the challenges for the surgeon in operating on patients with a high body mass index, frail individuals, and the elderly.

Our primary objective was to compare selected perioperative outcomes between the robot-assisted and laparoscopic surgery in patients with endometrial cancer, with particular emphasis on operative time, perioperative blood loss reflected by the difference between preoperative and postoperative hemoglobin levels.

In our study, we highlight complications occurring in both groups because they constitute an important indicator of perioperative safety, even though the absolute number of events was low.

Based on our findings, patients with endometrial cancer, particularly those who are older, obese, or burdened with comorbidities, may benefit from robot-assisted surgery, especially in terms of reduced blood loss, despite slightly longer operative times. Our manuscript is the first study describing such a large group in Poland. As mentioned earlier, it is a single-center study. Thanks to the existing centralization of oncological treatment in our region, gynecological oncologists have extensive surgical experience, and patients receive better medical care.

## 2. Materials and Methods

### 2.1. Study Sample Description

The study included 300 patients with endometrial cancer who underwent surgery at the Gynecological Oncology Department of the University Hospital in Rzeszow, Poland, using the da Vinci robotic system from December 2022 to December 2024, and 80 patients with endometrial cancer who underwent laparoscopic surgery from August 2021 to February 2023. Both groups underwent either a sentinel lymph node procedure or a lymphadenectomy.

Both surgical methods are minimally invasive and approved for the surgical treatment of endometrial cancer.

The patient’s well-being should always be paramount, and therefore, they should receive the best available medical care. Therefore, since the introduction of the robotic approach to our clinic, the vast majority of our patients with endometrial cancer have undergone this procedure. The vast majority of patients in the laparoscopic group underwent this procedure before the introduction of robotic procedures in our clinic, and some underwent surgery when the robotic approach was unavailable.

Because both methods are minimally invasive, the inclusion and exclusion criteria were the same for both groups. Inclusion criteria for both groups were as follows: histopathologically confirmed endometrial cancer after prior curettage of the cervical canal and uterine cavity; histopathological stage I and II according to FIGO 2009. Histopathological stage was assessed based on previously performed imaging studies, i.e., MRI of the reproductive organs, and computed tomography. Exclusion criteria were: FIGO stage >2; coexisting large uterine fibroids; coexisting ovarian tumors.

All patients consented to the study. The study was conducted in accordance with the approval of the Bioethics Committee of the Regional Medical Chamber dated 14 February 2019 (approval number: 24/B/2019).

### 2.2. Statistical Analysis

Statistical analysis of patients undergoing robotic surgery alone was performed using Statistica 13.3 (TIBCO Software Inc., Palo Alto, CA, USA, 2017). α = 0.05 was set as the threshold for statistical significance. Hypotheses regarding the normality of the distribution of the studied variables were tested using the Shapiro–Wilk test. None of the variables under the study had a distribution close to normal. Therefore, nonparametric tests were applied for further analyses. The analysis of variance was performed using the Kruskal–Wallis ANOVA test. Significantly different subgroups were tested using the Dunn’s post hoc test. Correlation analysis of the studied variables was performed using the Spearman rank correlation coefficient. Nominal variables were analyzed using the chi-square test with Yates’ continuity correction and Fisher test.

## 3. Results

### 3.1. Patients Who Underwent Only the Robotic Surgery

All patients undergoing robotic surgery were divided into age groups: 30–44 → Adult, 45–59 → Middle-aged, 60–74 → Senior, ≥75 → Elderly (Table 1).

**Figure 1:**
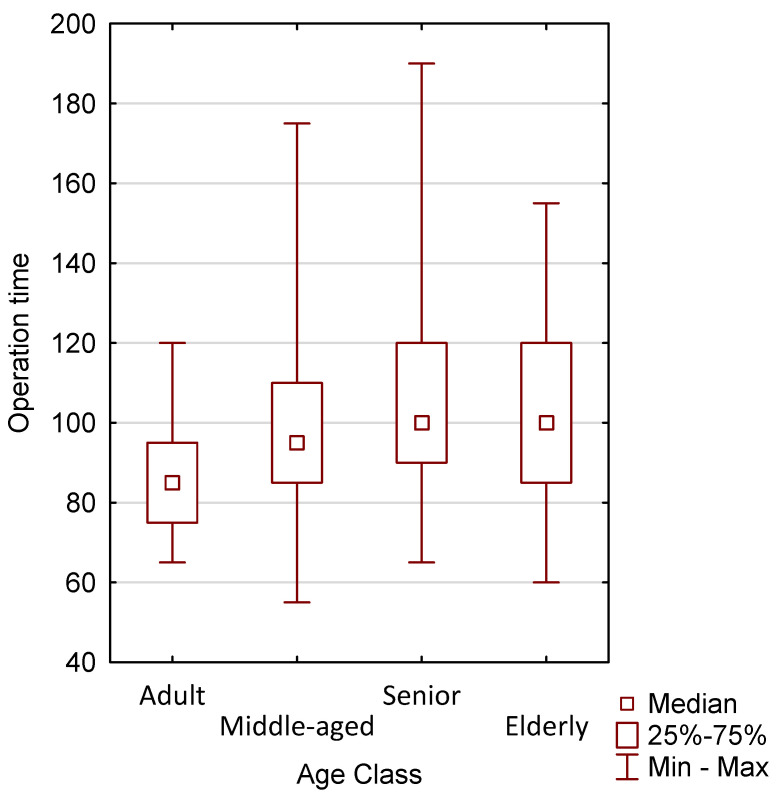
Graphical representation of operative times in patients undergoing robotic surgery according to age.

Among patients undergoing robotic surgery, statistical significance (*p* = 0.0025) was achieved when comparing the time of surgery (Table 2). A graphical comparison of the time of surgery in the above groups is presented in Figure 1.

**Figure 2:**
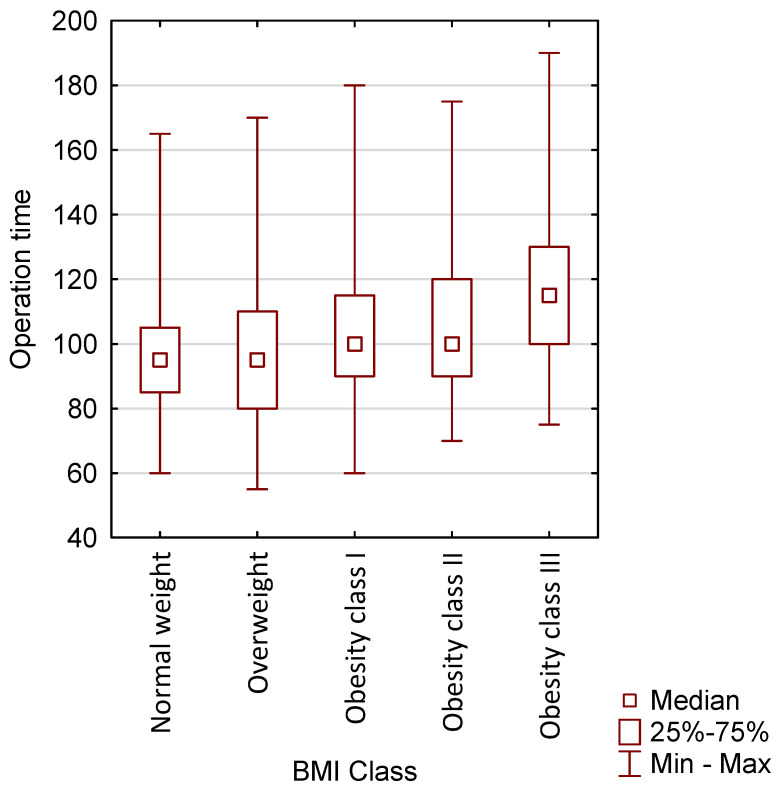
Graphical representation of operative times in patients undergoing robotic surgery according to BMI.

Patients undergoing robotic surgery were divided based on body weight into: 18.5–24.9 → Normal weight, 25.0–29.9 → Overweight, 30.0–34.9 → Obesity class I, 35.0–39.9 → Obesity class II, ≥40.0 → Obesity class III (Table 3). Using the Kruskal–Wallis test, the *p*-value for multiple comparisons was calculated, achieving statistical significance (*p* = 0.0002) (Table 4). A graphical comparison of operative times in the above groups is presented in Figure 2.

**Figure 3:**
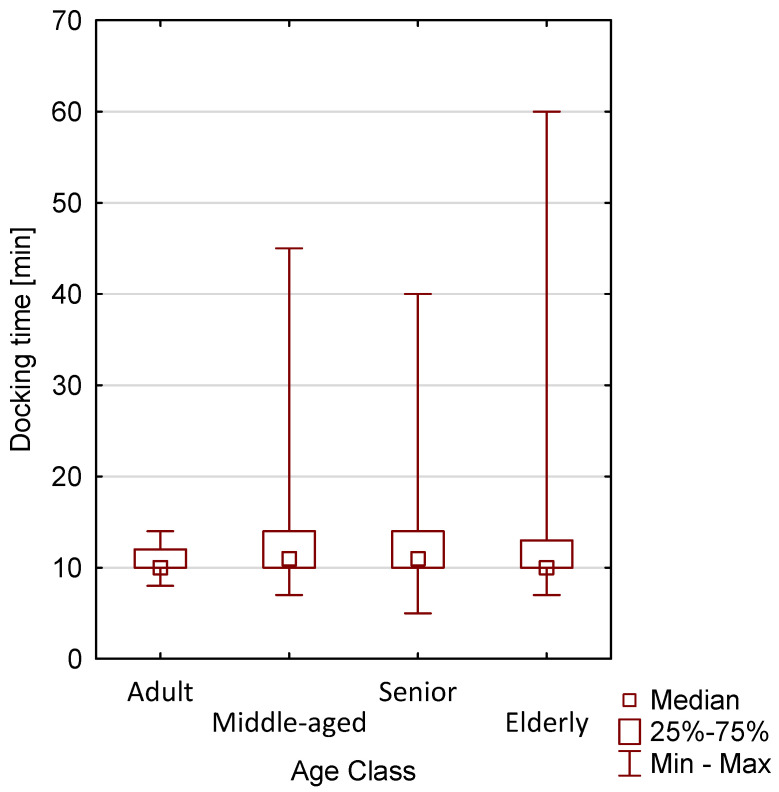
Graphical representation of the docking time in patients operated on using the robotic method depending on age.

**Figure 4:**
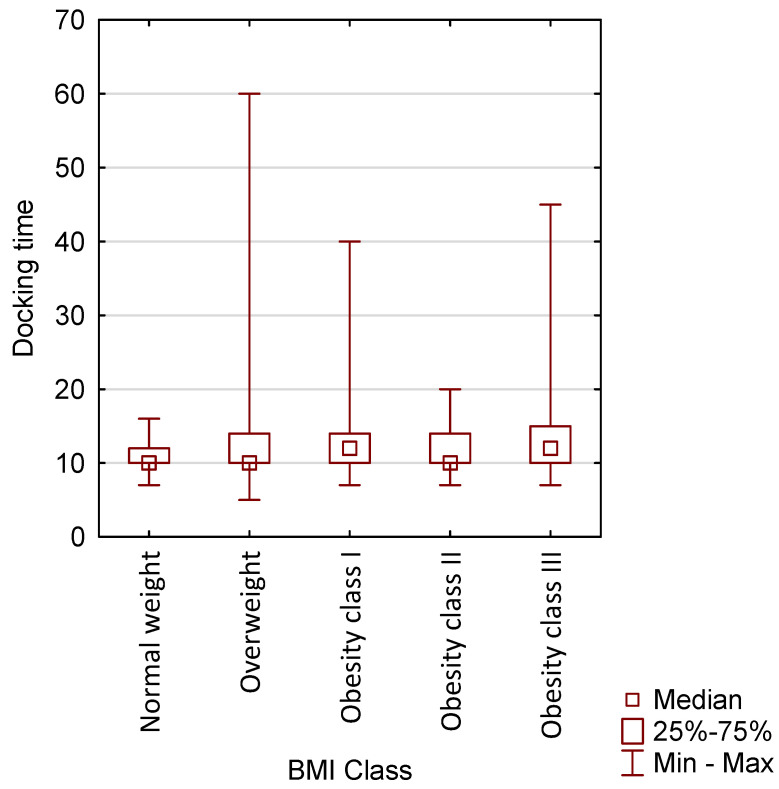
Graphical representation of the docking time in patients undergoing robotic surgery depending on BMI.

Among patients undergoing robotic surgery, docking time was also compared by age group (*p* = 0.3251) and by BMI (*p* = 0.0281). No statistically significant difference was observed across age groups, whereas a statistically significant association was found for BMI (Figure 3 and Figure 4).

### 3.2. Comparison of Patients Undergoing the Robotic and Laparoscopic Surgeries

During the second stage of the study, 300 robotic surgeries were compared to 80 laparoscopic surgeries (Table 5).

**Figure 5:**
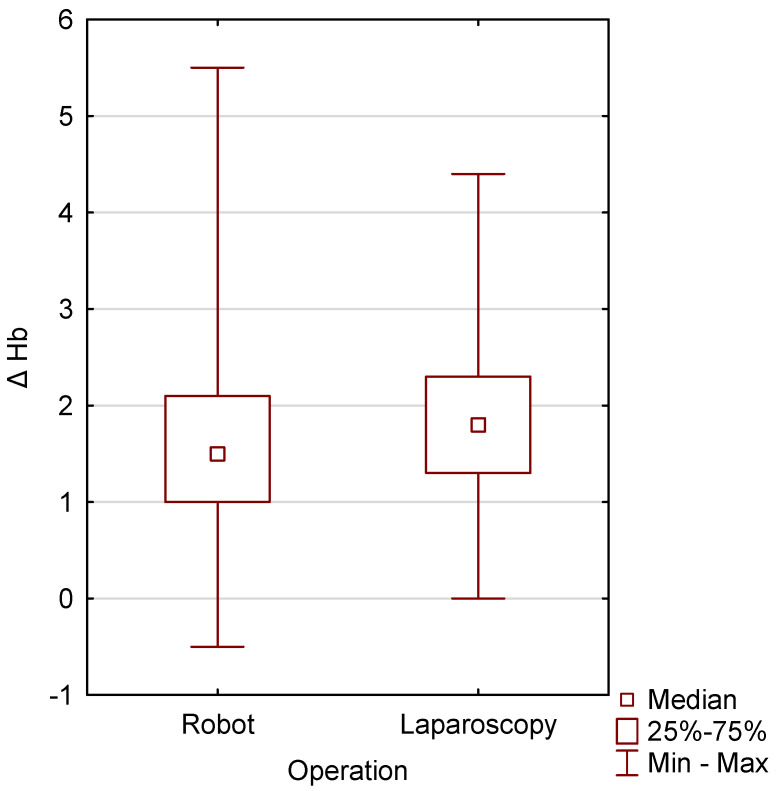
Graphical image showing the comparison of Delta Hb in two groups of patients, operated on using the robotic and laparoscopic methods.

The group of patients undergoing robotic surgery was compared with those undergoing laparoscopic surgery. Statistical significance was achieved for operative time (*p* = 0.0092), with the robotic surgery taking longer than the laparoscopic surgery. Statistical significance was also achieved in both groups for Delta Hb (*p* = 0.0124), with less blood loss occurring in the robotic group (Table 5, Figure 5).

Both surgical methods were compared in terms of intraoperative complications (Table 6). The absolute number of complications was low in both groups (2/300 in the robotic group vs. 3/80 in the laparoscopic group). Therefore, these findings should be interpreted with caution. Nevertheless, the observed distribution suggests a favorable perioperative safety profile of the robotic approach.

## 4. Discussion

Our study presents robot-assisted surgery on patients with endometrial cancer in FIGO stage I or II from 2009. This is the first study on such a large group conducted in Poland. In our region, oncology care is centralized. We operate on approximately 250 patients with endometrial cancer annually, which firstly, ensures that gynecologists are experienced surgeons, and secondly, patients experience fewer intraoperative and postoperative complications. In our group undergoing da Vinci-assisted surgery, we observed only two complications: intestinal wall damage, which was repaired with sutures. We had no cases of conversion to laparotomy. According to the literature, centralized care, particularly in gynecological oncology, improves patient outcomes [15].

Lindfors et al. compared surgical outcomes and survival after primary robotic surgery with open surgery (laparotomy) in obese women with endometrial cancer. The study included 217 patients (131 robotic and 86 open procedures) with a BMI ≥ 30 kg/m^2^. The investigators demonstrated that the robotic surgery in obese women with EC provided similar long-term survival and disease-free survival compared with the open surgery, with significantly fewer complications, less estimated blood loss, shorter operating times, and shorter hospital stays. The researchers recorded six conversions in the robotic group. The conversion rate was 4.6%. Reasons included: difficulty with oxygen saturation during the Trendelenburg position, unexpected pelvic freezing, difficult surgical access to the pelvis, uterine bleeding obstructing surgical vision, bladder trauma, and one conversion due to technical issues with the da Vinci system. [13]. In our study, 165 patients in the robotic group had BMI > 30, but we recorded only two intestinal complications. According to our data, the length of hospitalization ranged from 4 to 6 days in both study groups, so we did not use this variable in comparisons between the two groups. Extended hospitalizations were often due to days off.

The introduction of the robotic surgery in Denmark for the treatment of early-stage endometrial cancer was associated with improved survival regardless of age, body mass index, ASA score, comorbidities, smoking status, socioeconomic status, and histopathological risk. This was a multicenter, prospective study that included a large number of patients—2563—who underwent robotic-assisted surgery. The researchers observed no significant difference in survival between laparoscopic and robotic-assisted surgery [16].

A large meta-analysis by Ran et al. included 4420 patients who underwent robotic, laparoscopic, and conventional (open) surgery. In this study, the authors demonstrated that robotic surgery is a safer and more reliable method than laparoscopy and laparotomy in patients with endometrial cancer. Robotic surgery is associated with significantly less blood loss than laparoscopy and laparotomy and fewer conversions. However, the researchers observed a higher rate of complications with robotic surgery than with laparoscopy [17].

Kakkos et al. conducted a retrospective analysis of 598 women with clinical stage I EC who underwent robotic surgery in Belgium. The researchers found that the conversion rate to laparotomy was low (0.8%), and the mean postoperative Comprehensive Complication Index (CCI) score was 3.4. Importantly, the perioperative complication rate did not differ between age groups. The researchers also found that disease-free survival was significantly lower in patients over 75 years of age compared to those under 65 years of age. It is also significant that an increase in BMI had no effect on perioperative complications, the conversion rate to laparotomy, postoperative hospital stay, CCI score, or disease-free survival. The study included patients operated on in five centers and all of them had FIGO stage I EC. [14]. In our study, although all patients were operated on at a single center, we confirmed these observations in patients undergoing robot-assisted surgery.

A meta-analysis of 51 observational studies by Cusimano et al. included 10,800 patients diagnosed with endometrial cancer and obesity (BMI 31.0–56.3 kg/m^2^). The authors compared robot-assisted surgery with laparoscopy. The need for transfusion occurred in 2.1% of patients undergoing the robot-assisted procedures, whereas in the laparoscopic group it reached 2.8%. They also highlighted an almost threefold lower risk of visceral or major vascular injury with robot-assisted surgery (1.2% vs. 3.5%). Regarding thromboembolic complications, both approaches were associated with a similar risk of 0.5%. Cusimano et al. also evaluated conversion rates with increasing BMI. In both groups, the most common reason for conversion was inadequate exposure of the operative field due to peritoneal adhesions or visceral obesity. Among patients with BMI > 30, conversion to laparotomy occurred in 5.5% of robot-assisted cases and 6.5% of laparoscopic cases. In patients with morbid obesity (BMI > 40), the conversion rate for the robot-assisted surgery was nearly twofold lower—3.8% in the robotic group compared with 7.0% in the laparoscopic group [18]. Among our patients, we did not observe any conversion in either the laparoscopic or robot-assisted surgery groups.

In our cohort, the number of intraoperative complications was very low in both groups. Therefore, although the observed pattern was consistent with the international literature, complication-related findings should be interpreted cautiously and viewed primarily as supportive perioperative observations rather than definitive evidence of the superiority of one technique over the other.

The low number of events also limits the applicability of the Clavien-Dindo (CD) classification. This system provides a standardized, five-level assessment of surgical complications based on the degree of therapeutic intervention required to manage them.

Furthermore, we fully acknowledge that the imbalance in group sizes and the different time periods of patient inclusion constitute a limitation of our study and may introduce bias related to patient selection, surgical experience, and the institutional learning curve. However, it is important to emphasize that, in the current era, assembling a sufficiently large cohort of patients undergoing purely laparoscopic procedures has become increasingly challenging due to the widespread adoption of robotic surgery. This shift in clinical practice inevitably leads to disproportionate group sizes when retrospective comparisons are performed across different time periods.

As mentioned at the beginning of this chapter, centralization of care, particularly in gynecological oncology, is crucial for patient care and improves treatment outcomes. Centralization may also have certain advantages in the future, specifically in telesurgery, which allows surgeries to be performed on patients in peripheral hospitals by experienced surgeons, as emphasized by Pavone et al. [15].

Computer-assisted intraoperative data collection, information processing, and decision support systems hold an enormous promise. Technologies such as virtual reality (VR) and AR are becoming increasingly common in everyday life and are gradually being incorporated into MIS systems. The next steps, according to Pavone et al., aim to introduce experimental techniques in robotic surgery that will enable intraoperative microscopic visualization, ideally detecting small-volume metastases and improving the sensitivity of frozen sections in gynecologic oncology. According to Pavone et al., future prospective studies will focus on integrating robotic platforms with artificial intelligence systems, image-guided surgery, and overcoming physical limitations through telesurgery [15].

## 5. Conclusions

Based on the analysis of our single-center cohort including 300 patients who underwent robot-assisted surgery and 80 patients who underwent laparoscopic surgery, robot-assisted surgery was associated with lower blood loss, whereas operative time remained shorter with laparoscopy. The number of intraoperative complications was low in both groups, and although the observed pattern favored the robotic approach, these findings should be interpreted with caution due to the small absolute number of events. We also observed that prolonged robotic operative time occurred particularly in older patients and those with higher BMI, which may be relevant when planning surgical procedures. Our findings are consistent with reports from the international literature and support the clinical value of robotic techniques, especially in selected higher-risk patient populations. Further studies are warranted to better define the patient groups deriving the greatest benefit and to optimize surgical management strategies.

## Figures and Tables

**Table 1:** Characteristics of the group of patients operated on using the robotic method, taking into account the time of operation and preoperative and postoperative Hb (N = 300).

ADULT							
	N	%	Median	Minimum	Maximum	Lower Quartile	Upper Quartile
Operation time	10	100.00	85.00	65.00	120.00	75.00	95.00
Preoperative Hb	10	100.00	13.40	9.40	15.00	11.10	14.20
Postoperative Hb	9	90.00	12.00	8.70	13.60	9.80	12.90
Middleaged							
Operation time	89	100.00	95.00	55.00	175.00	85.00	110.00
Preoperative Hb	89	100.00	13.30	9.30	16.10	12.30	14.10
Postoperative Hb	85	95.51	11.90	8.00	14.60	11.00	12.70
Senior							
Operation time	139	100.00	100.00	65.00	190.00	90.00	120.00
Preoperative Hb	139	100.00	13.90	9.60	16.40	13.30	14.60
Postoperative Hb	137	98.56	12.30	8.90	14.80	11.60	13.00
Elderly							
Operation time	62	100.00	100.00	60.00	155.00	85.00	120.00
Preoperative Hb	61	98.39	13.30	9.60	16.30	12.40	14.00
Postoperative Hb	60	96.77	11.40	7.70	14.80	10.50	12.40

Division of patients undergoing robotic surgery into age groups: 30–44 → Adults, 45–59 → Middle age, 60–74 → Seniors, ≥75.

**Table 2:** Comparison of patients undergoing robotic surgery, by age group, in terms of operative time (N = 300).

Operation Time	Adult, R: 82.950	Middle-Aged, R: 134.69	Senior, R: 167.10	Elderly, R: 146.88
Adult		0.4424	0.0183	0.1834
Middle-aged	0.4425		0.0355	1.0000
Senior	0.0183	0.0355		0.7615
Elderly	0.1834	1.0000	0.7615	

Among patients undergoing robotic surgery, statistical significance was achieved (*p* = 0.0025) when comparing the duration of surgery; *p* value for multiple comparisons (bilateral); The Kruskal–Wallis test; *p* = 0.0025.

**Table 3:** Division of patients operated on using the robotic method in terms of BMI (N = 300).

Normal Weight	N	%	Median	Minimum	Maximum	Lower Quartile	Upper Quartile
Operation time	44	100.00	95.00	60.00	165.00	85.00	105.00
Preoperative Hb	44	100.00	13.40	10.00	15.90	12.05	14.60
Postoperative Hb	43	97.73	11.90	9.30	14.80	10.80	12.80
Variable: Overweight							
Operation time	86	100.00	95.00	55.00	170.00	80.00	110.00
Preoperative Hb	86	100.00	13.50	9.30	16.30	12.50	14.10
Postoperative Hb	86	100.00	11.70	8.00	14.00	10.80	12.60
Obesity class I							
Operation time	79	100.00	100.000	60.00	180.00	90.00	115.00
Preoperative Hb	78	98.73	13.70	9.60	15.80	12.90	14.20
Postoperative Hb	74	93.67	12.15	7.70	14.40	11.60	14.90
Obesity class II							
Operation time	57	100.00	100.00	70.00	175.00	90.00	120.00
Preoperative Hb	57	100.00	14.00	10.90	16.40	13.30	14.60
Postoperative Hb	55	96.49	12.40	8.50	14.80	11.20	13.00
Obesity class III							
Operation time	29	100.00	115.00	75.00	190.00	100.00	130.00
Preoperative Hb	29	100.00	13.50	9.40	16.10	12.70	14.00
Postoperative Hb	28	96.55	12.20	8.70	14.50	10.85	12.65

Division of patients undergoing robotic surgery based on body weight into: 18.5–24.9 → Normal weight, 25.0–29.9 → Overweight, 30.0–34.9 → Obesity class I, 35.0–39.9 → Obesity class II, ≥40.0 → Obesity class III.

**Table 4:** Comparison of robotic surgery patients, taking into account BMI, in terms of operative time (N = 295).

Operation Time	Obesity Class II, R: 164.68	Obesity Class I, R: 150.57	Overweight, R: 129.65	Obesity Class III, R: 201.41	Normal Weight, R: 122.44
Obesity class II		1.0000	0.1618	0.5906	0.1360
Obesity class I	1.0000		1.0000	0.0608	0.7963
Overweight	0.1618	1.0000		0.0009	1.0000
Obesity class III	0.5906	0.0605	0.0009		0.0011
Normal weight	0.1360	0.7963	1.0000	0.0011	

Patients undergoing robotic surgery were divided based on body weight. The Kruskal–Wallis test was used to calculate the *p*-value for multiple comparisons, achieving statistical significance (*p* = 0.0002); *p* value for multiple comparisons (bilateral); The Kruskal–Wallis test; *p* = 0.0002.

**Table 5:** Characteristics of two groups of patients operated on: robotic and laparoscopic.

Variable		Robot-Assisted Surgery (N = 300)	Laparoscopic Surgery (N = 80)	*p*-Value
Operation time	Mean ± SD Median (Q1–Q3) Min–Max	103.44 ± 23.66 100.00 (85.00–115.00) 55.00–190.00	96.88 ± 2 6.26 90.00 (75.00–115.00) 50.00–160.00	0.0092
BMI	Mean ± SD Median (Q1–Q3) Min–Max	46.26 ± 2.66 31.22 (27.24–35.92) 19.33–45.91	32.82 ± 5.98 32.75 (28.08–36.53) 21.34–50.21	0.1397
Delta Hb	Mean ± SD Median (Q1–Q3) Min–Max	1.55 ± 0.85 1.50 (1.00–2.10) 0.50–5.50	1.77 ± 0.83 1.80 (1.30–2.30) 0.00–4.40	0.0124

Comparison of the robotic and laparoscopic groups. Significance was achieved for operative time (*p* = 0.0092), with the robotic procedure taking longer than the laparoscopic procedure. Delta Hb was also significant in both groups (*p* = 0.0124), with the robotic group experiencing less blood loss. The Mann–Whitney U test.

**Table 6:** Summary table, divided into two parts, showing the number of complications.

	Without Complications	Complications	*p*-Value
Robot (N = 300)			
% column	79.47%	40.00%	
% row	99.33%	0.67%	
Laparoscopic surgery (N = 80)			0.0605
% column	20.53%	60.00%	
% row	96.25%	3.75%	

Comparison of complications in the robotic and laparoscopic groups achieved a borderline statistical significance (*p* = 0.0605). Fisher test.

## Data Availability

The data presented in this study are available on request from the corresponding authors.

## References

[1] Crosbie E.J., Kitson S.J., McAlpine J.N., Mukhopadhyay A., Powell M.E., Singh N. (2022). Endometrial cancer. Lancet.

[2] Bray F., Ferlay J., Soerjomataram I., Siegel R.L., Torre L.A., Jemal A. (2018). Global cancer statistics 2018: GLOBOCAN estimates of incidence and mortality worldwide for 36 cancers in 185 countries. CA Cancer J. Clin..

[3] Renehan A.G., Tyson M., Egger M., Heller R.F., Zwahlen M. (2008). Body-mass index and incidence of cancer: A systematic review and meta-analysis of prospective observational studies. Lancet.

[4] Nevadunsky N.S., Van Arsdale A., Strickler H.D., Moadel A., Kaur G., Levitt J.B., Girda E., Goldfinger M., Goldberg G.L., Einstein M.H. (2014). Obesity and age at diagnosis of endometrial cancer. Obstet. Gynecol..

[5] Friedenreich C.M., Neilson H.K., Lynch B.M. (2010). State of the epidemiological evidence on physical activity and cancer prevention. Eur. J. Cancer.

[6] Visser N.C.M., Reijnen C., Massuger L.F., Nagtegaal I.D., Bulten J., Pijnenborg J.M. (2017). Accuracy of Endometrial Sampling in Endometrial Carcinoma: A Systematic Review and Meta-analysis. Obstet. Gynecol..

[7] Lin M.Y., Dobrotwir A., McNally O., Abu-Rustum N.R., Narayan K. (2018). Role of imaging in the routine management of endometrial cancer. Int. J. Gynecol. Obstet..

[8] Faria S.C., Devine C.E., Rao B., Sagebiel T., Bhosale P. (2019). Imaging and Staging of Endometrial Cancer. Seminars in Ultrasound, CT and MRI.

[9] Pecorelli S. (2009). Revised FIGO staging for carcinoma of the vulva, cervix, and endometrium. Int. J. Gynecol. Obstet..

[10] Liu T., Tu H., Li Y., Liu Z., Liu G., Gu H. (2019). Impact of Radical Hysterectomy Versus Simple Hysterectomy on Survival of Patients with Stage 2 Endometrial Cancer: A Meta-analysis. Ann. Surg. Oncol..

[11] Galaal K., Bryant A., Fisher A.D., Al-Khaduri M., Kew F., Lopes A.D. (2018). Laparoscopy versus laparotomy for the management of early stage endometrial cancer. Cochrane Database Syst. Rev..

[12] Dinoi G., Ghoniem K., Murad M.H., Segarra-Vidal B., Zanfagnin V., Coronado P.J., Kyrgiou M., Perrone A.M., Zola P., Weaver A. (2023). Minimally Invasive Compared With Open Surgery in High-Risk Endometrial Cancer: A Systematic Review and Meta-analysis. Obstet. Gynecol..

[13] Lindfors A., Heshar H., Adok C., Sundfeldt K., Dahm-Kähler P. (2020). Long-term survival in obese patients after robotic or open surgery for endometrial cancer. Gynecol. Oncol..

[14] Kakkos A., Eecke C.V., Ongaro S., Traen K., Peeters F., Van Trappen P., Laenen A., Despierre E., Van Nieuwenhuysen E., Vergote I. (2021). Robot-assisted surgery for women with endometrial cancer: Surgical and oncologic outcomes within a Belgium gynaecological oncology group cohort. Eur. J. Surg. Oncol..

[15] Pavone M., Goglia M., Rosati A., Innocenzi C., Bizzarri N., Seeliger B., Mascagni P., Ferrari F.A., Forgione A., Testa A.C. (2025). Unveiling the real benefits of robot-assisted surgery in gynaecology: From telesurgery to image-guided surgery and artificial intelligence. Facts Views Vis. Obgyn.

[16] Jørgensen S.L., Mogensen O., Wu C.S., Korsholm M., Lund K., Jensen P.T. (2019). Survival after a nationwide introduction of robotic surgery in women with early-stage endometrial cancer: A population-based prospective cohort study. Eur. J. Cancer.

[17] Ran L., Jin J., Xu Y., Bu Y., Song F. (2014). Comparison of robotic surgery with laparoscopy and laparotomy for treatment of endometrial cancer: A meta-analysis. PLoS ONE.

[18] Cusimano M.C., Simpson A.N., Dossa F., Liani V., Kaur Y., Acuna S.A., Robertson D., Satkunaratnam A., Bernardini M.Q., Ferguson S.E. (2019). Laparoscopic and robotic hysterectomy in endometrial cancer patients with obesity: A systematic review and meta-analysis of conversions and complications. Am. J. Obstet. Gynecol..

